# OBM-RFEcv: An adaptive ensemble model for monitoring key growth indicators of Gerbera using multi-spectral image fusion features

**DOI:** 10.1371/journal.pone.0322851

**Published:** 2025-05-20

**Authors:** Xinrui Wang, Yingming Shen, Peng Tian, Mengyao Wu, Zhaowen Li, Jiawei Zhao, Jihong Sun, Ye Qian

**Affiliations:** 1 College of Big Data, Yunnan Agricultural University, Kunming, Yunnan, China; 2 International Cooperation Office, Yunnan Provincial Academy of Science and Technology, Kunming, Yunnan, China; 3 College of Agronomy and Biotechnology, Yunnan Agricultural University, Kunming, Yunnan, China; 4 Yunnan Key Laboratory of Computer Technologies Application, Kunming University of Science and Technology, Kunming, Yunnan, China; 5 The Key Laboratory for Crop Production and Smart Agriculture of Yunnan Province, Yunnan Agricultural University, Kunming, Yunnan, China; Shahjalal University of Science and Technology, BANGLADESH

## Abstract

This study aims to address the challenge of monitoring Plant Height (PH), SPAD, Leaf Area Index (LAI), and Above-Ground Biomass (AGB) in Gerbera under greenhouse cultivation conditions. We initially gathered multi-spectral images and corresponding ground truth data of these parameters at various growth stages using a low-altitude UAV. From the collected images, we derived five Vegetation Indices (VIs): NDVI, GNDVI, LCI, NDRE, and OSAVI, and extracted their textural features as fusion features. An adaptive ensemble model, OBM-RFEcv, was then developed by integrating six base models (Linear Regression, Decision Tree Regressor, Random Forest Regressor, XGBoost Regressor, and Support Vector Regressor) with Recursive Feature Elimination (RFE) to predict the key growth indicators. The results indicate that the OBM-RFEcv model outperforms the other models when using the fusion of the five VIs, particularly in the test dataset, where it achieved the highest accuracy for PH (NDVI), SPAD (GNDVI), LAI (GNDVI), and AGB (NDRE) with R^2^ values of 0.92, 0.90, 0.89, and 0.93, respectively. The root mean square error (RMSE) values were 0.04, 0.07, 0.08, and 0.07, respectively, showing improvements over the best individual model by 0.01, 0.03, 0.01, and 0.09 in R^2^, and reductions in RMSE by 0.01, 0.07, 0.08, and 0.03, respectively. These findings confirm that the OBM-RFEcv model, based on multi-spectral image fusion, effectively monitors key growth indicators in Gerbera, providing a non-invasive and precise method for greenhouse crop monitoring.

## Introduction

Gerbera, commonly known as the African daisy, is one of the world’s top five cut flowers [1]. In China, Gerbera has gained popularity as a cut flower due to its high yield, extensive cultivation, and significant economic returns [1,2]. To ensure robust growth and high-quality flower production, Gerbera requires stringent environmental controls [3]. Key indicators of crop health, such as Plant Height (PH), SPAD, Leaf Area Index (LAI), and Above-Ground Biomass (AGB), are essential for assessing the developmental status of Gerbera [4–6]. Accurate and efficient monitoring of these indicators in greenhouse settings is vital for effective field management [4].

Traditional methods for measuring PH, SPAD, LAI, and AGB include field measurements or laboratory analyses [7]. These methods, particularly those for LAI and AGB, often require destructive sampling at different growth stages, which is both time-consuming and labor-intensive [8,9]. Therefore, there is a pressing need for developing a method that can provide rapid, non-destructive, and accurate monitoring of these critical parameters during the growth cycle of greenhouse crops.

In the context of crop growth monitoring, the most common traditional approach involves the use of tools like DSSAT and WOFOST to construct crop growth models. For example, Mulla et al. [10] calibrated the DSSAT CERES-Maize and CROPGRO-PFM models with high nitrogen application rates to simulate intermediate wheat grass and predict yield, biomass, soil water balance, and soil nitrogen balance under medium and low nitrogen application rates. Wang et al. [11] used the WOFOST model to evaluate the impact of varying model parameters on goji berry yield under different climatic conditions. Tang et al. [12] developed a WOFOST-Chili model to quantify dry matter production as a function of fertilizer management, climate, and soil conditions. Amiri [13] employed the WOFOST model to simulate maize growth, LAI, biomass, grain yield, yield gap, and soil moisture under both irrigation and rainfall conditions, evaluating different growth periods and the entire growing season. Raghavendra et al. [14] used the DSSAT model to estimate potential soybean yields in different regions of India, validating the model under various agricultural ecological conditions and deriving the correlation between soybean yield and different agricultural ecological conditions. These studies have improved crop cultivation practices and and contributed to monitoring of the crop growth process. However, traditional crop growth models rely on a large number of environmental parameter inputs, including meteorological conditions, soil properties, etc. The collection of these data often requires complex equipment and professional technology, leading to high data acquisition costs and low efficiency. Moreover, most of the aforementioned studies have focused on field crops, with relatively few studies on horticultural crops or crops in special environments, which limits the application scope of the models. By using spectral sensors mounted on Unmanned Aerial Vehicles (UAVs), it is possible to efficiently obtain real-time image data of field crops, not only improving the efficiency of data collection but also expanding the monitoring range, which contributes to more accurately assessing crop growth status. Optimizing model parameters through machine learning algorithms can improve the prediction accuracy of the models while reducing the heavy reliance on specific regional and crop data.

In recent years, satellite and Unmanned Aerial Vehicle (UAV)-based multi-spectral remote sensing technologies have gained popularity for monitoring crop growth processes. These technologies primarily use satellite remote sensing platforms to monitor large areas of crop growth and estimate key crop indicators. For instance, Sun et al. [15] used time-series Sentinel-1A satellite data to extract the Dual-Polarization Radar Vegetation index (DPRVI) and integrated it with empirical regression models to invert sugarcane plant height, effectively capturing the changes in plant height during the early growth stage. Ma et al. [16] assimilated the LAI derived from Sentinel-2 satellite data into the SAFY (Simple Algorithm for Yield Estimation crop growth model for wheat yield estimation. Integrating remote sensing data into crop growth models aids in estimating LAI, biomass, and yield. Gupta et al. [17] used LISS-IV satellite data to spatially model SPAD (Soil-Plant Analysis Development) values, enabling effective monitoring of chlorophyll content distribution in agricultural areas. Tang et al. [18] built and validated a customized model for grassland biomass in the Yellow River source region using AGB ground truth data, environmental variables, and vegetation indices, analyzing the spatiotemporal distribution and dynamic trends of grassland biomass over 20 years. Yang et al. [19] used multi-source, multi-angle image data from Landsat-5 and small satellite multispectral sensors to construct a LAI inversion model combining radiative transfer models, mean clustering, and artificial neural networks, improving the average accuracy of LAI inversion by approximately 30% compared to single-angle data. However, in current research, satellite remote sensing images mostly have lower resolution, which limits the ability to accurately monitor small plots or orchards. Moreover, they predominantly focus on specific crop growth indicators, lacking comprehensive monitoring methods that can fully reflect the crop growth status and its environmental impact factors. Combining UAVs with multispectral sensors for low-altitude flights can acquire higher-resolution data, enabling more precise monitoring of crop growth conditions within small plots or specific agricultural areas. Utilizing machine learning or deep learning algorithms to analyze integrated features allows for a deeper assessment of crop growth status while also enhancing the diversity and accuracy of monitoring indicators.

Concurrently, UAV-based multi-spectral remote sensing technology, with its advantages of fast, real-time, and high-resolution imaging, has become a crucial tool in agricultural remote sensing. Zu et al. [20] used multi-spectral images obtained by UAVs to build machine learning and Convolutional Neural Network (CNN) models for estimating winter wheat LAI, demonstrating that the CNN model had the highest estimation accuracy. Ji et al. [21] constructed 26 multi-spectral vegetation indices combined with ground-measured SPAD values of wheat, using four machine learning algorithms to develop SPAD value estimation models for different stages of wheat. Xie et al. [22] used UAV multi-spectral data, combined with vegetation indices and texture features, to predict the SPAD value of lychee fruits during the growth and autumn sprouting periods. Prakriti Sharma et al. [23] used multi-spectral data before and after oat heading obtained by UAVs, combined with four machine learning algorithms, to construct oat biomass estimation models, exploring the potential for estimating AGB in oat breeding nurseries.

Machine learning methods can enhance the prediction accuracy of crop growth traits through data fusion based on UAV multi-spectral images [24]. Yu et al. [25] developed a simple and effective LAI estimation method, using Kalman Filtering (KF) to integrate the estimation results of multiple vegetation index models for precise LAI monitoring during different growth stages of rice. Zhou et al. [26] used machine learning algorithms to develop Canopy Cover Density (CCD), Leaf Chlorophyll Density (LCD), and LAI estimation models by integrating spectral features, texture features, and wavelet coefficient features obtained from multi-spectral images. Ma et al. [27] combined vegetation indices (VIs), color indices (CIs), and texture features to improve the accuracy of assessing crop disease severity from UAV images. Wen-jing [28] formed a multi-source feature dataset using UAV spectral features, thermal features, structural features, and chlorophyll content data, building chlorophyll content estimation models for corn at the jointing, tassel, and large tassel stages using four machine learning algorithms, proving that multi-source feature fusion and stacked ensemble learning can effectively improve the accuracy of chlorophyll content estimation. Naveed et al. [29] used a Predictive Coding/Biased Competition-Divisive Input Modulation (PC/BC-DIM) neural network to determine multi-spectral fusion saliency maps for predicting prominent crops and detecting weeds, achieving higher average accuracy compared to unfused data.

In current research, although machine learning methods have been used to fuse data from UAV multispectral images, thereby improving the prediction accuracy of crop growth traits, most models and methods lack an adaptive mechanism. When faced with changes in environmental conditions, crop types, or growth stages, they cannot automatically adapt to new situations. Despite the fact that data fusion can improve the prediction accuracy of crop growth traits, these methods often involve complex algorithms and require substantial computational resources, increasing the difficulty and cost of practical applications. By simplifying data fusion strategies, reducing the complexity of data processing, and eliminating unnecessary complex computational steps, while incorporating adaptive mechanisms into machine learning models, it is possible to enable these models to automatically adjust their structure based on real-time acquired data. This allows them to adapt to different environmental conditions and crop types.

The aforementioned studies have utilized various methods, including crop growth models, satellite remote sensing, UAV multispectral remote sensing technology, and multispectral image feature fusion, to achieve the inversion, estimation, and prediction of key characteristic values during the crop growth process. Currently, researchers mainly focus on field crops, with multispectral image data primarily collected via high-altitude UAVs. There is very limited research reported on economic crops grown in greenhouses. Moreover, in terms of feature fusion, it often involves complex calculations or simply stacking different features, and there is also limited research on ensemble methods for regression models. Therefore, the main objective of this study is to propose a feature fusion method based on extracting texture features from vegetation index images using multispectral images captured at low altitudes within greenhouse environments, combined with ground-measured data such as PH, SPAD, LAI, and AGB. By employing an adaptive ensemble model for model and feature variable selection, we aim to improve the monitoring accuracy of growth indicators for Gerbera at different growth stages and construct a monitoring model for the growth process of Gerbera. The specific objectives are: (i) to evaluate the performance of the adaptive ensemble model with model and feature variable selection in multi-indicator monitoring during the growth process of Gerbera; (ii) to assess the application potential of the proposed feature fusion method that extracts texture features from different vegetation index images in monitoring key growth indicators of Gerbera. This provides a reference for using multispectral UAVs for low-altitude monitoring of economic crops grown in greenhouses, further promoting the application of multispectral technology in the growth models of economic crops.

### Paper structure

The structure of the other sections of this paper is as follows: it is divided into four sections—Materials and methods, Results, Discussion, and Conclusion. Materials and methods mainly introduce the experimental site and planting conditions for the study subject Gerbera, followed by the methods for collecting growth indicators and multispectral image data of Gerbera and the data processing procedures. It concludes with the construction process of the adaptive ensemble model OBM-RFEcv and the evaluation metrics used for the models. In the Results section, it showcases the application effects of the OBM-RFEcv model in monitoring PH, SPAD, LAI, and AGB of Gerbera, comparing the performance between base models and the OBM-RFEcv model. Through charts and data analysis, it reveals the relationships among various parameters and their impacts on Gerbera’s growth status, while also incorporating SHAP visualizations to analyze the interpretability of the OBM-RFEcv model. In the Discussion section, based on the previous results, it delves into the significance of feature fusion in monitoring Gerbera growth indicators, comparing and analyzing these findings with existing literature. It also discusses in-depth the reasons behind the superior performance of the OBM-RFEcv model, its limitations, and potential directions for future research. In the final Conclusion section, it summarizes the main conclusions based on the research findings, emphasizing the practical value of the OBM-RFEcv model, and offers insights into potential future development trends.

## Materials and methods

### Experimental site and growing conditions

**Fig 1:**
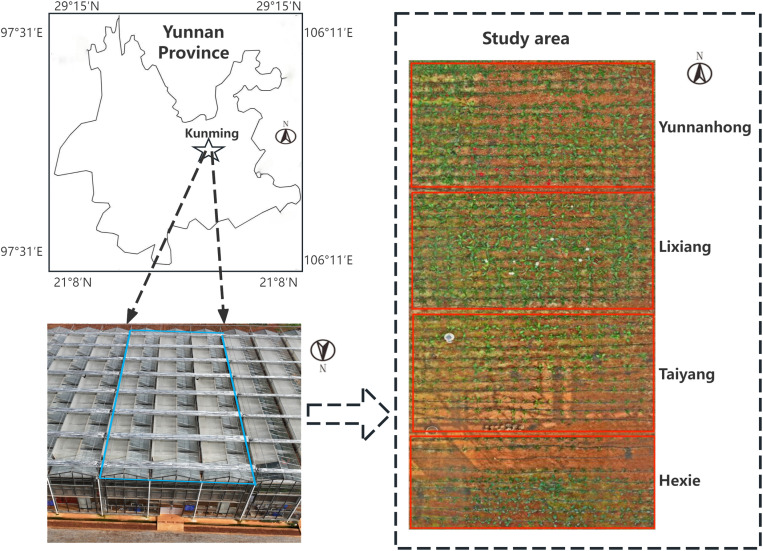
Experimental site and study area.

The experimental area is located in the greenhouse of zone 2–22 at the New Agricultural Science Comprehensive Practical Teaching Base of Yunnan Agricultural University (longitude 102°45’49’‘E to 102°45’50’‘E, latitude 25°13’61’‘N to 25°13’63’‘N, see Fig 1). The dimensions of the greenhouse are approximately 35 meters in length, 10 meters in width, and 5 meters in internal height. Four varieties of Gerbera, namely “*Yunnanhong*”, “*Lixiang*”, “*Taiyang*”, and “*Hexie*”, were cultivated. The layout of the planting areas is illustrated in Fig 1. The planting period was from December 20, 2023, to April 10, 2024. Each variety was planted in an area of approximately 40 square meters, with the entire experimental plot covering about 160 square meters. The planting density was 6–9 plants per square meter, and the average plant spacing was 40 centimeters. The research area’s average growing environment included a carbon dioxide concentration of 313 ppm, soil moisture content of 16.18%, soil temperature of 21.87°C, air temperature of 22°C, air humidity of 70.99%, and solar radiation of 3.57 kilolux (kLx). To ensure healthy growth of Gerbera, organic fertilizer was applied once every 20 days, watering was conducted twice a week, and weeding as well as removal of yellow leaves were performed once a week during the planting period of Gerbera.

### Data collection

Data collection was conducted during the growth, early flowering, and full bloom stages of the Gerbera. The data were collected from January 20, 2024, to April 10, 2024, with a total of seven collections: three during the growth stage, two during the early flowering stage, and two during the full bloom stage. The ground data collection for Gerbera growth indicators primarily included PH, LAI, SPAD, and AGB.

First, the experimental area was divided into smaller sections, with each variety being divided into six relatively uniform small sections, resulting in a total of 24 small sections. In each small section, four evenly growing plants were randomly selected as samples for each data collection. After measuring the growth indicators of the four sample plants, the average value was taken as one set of sample data. The methods for collecting and measuring the various growth indicators are as follows:

#### PH measurement.

The PH of Gerbera was measured as the height of the highest leaf in its natural state relative to the ground. A height ruler was used, held vertically to the ground, for the measurement.

#### SPAD measurement.

For the collection of SPAD data, the SPAD-502 Plus instrument was used. Functional leaves with a width greater than 3 cm or a length greater than 5 cm were selected from each sample plant, and their SPAD values were measured. The average value of these measurements was taken as the SPAD value for that sample plant.

#### LAI measurement.

The specific leaf weight method, which uses the ratio of unit leaf area to dry leaf weight to obtain the leaf area index, was employed. This method is simple and relatively accurate, often used for comparative measurements of crops, although the measurement process is complex. To calculate LAI, four sample plants from each small section (A) (area approximately 6 m²) were collected and measured in the laboratory. Before measurement, the total number of plants (N) in the sampling area was recorded. All expanded green leaves were removed from the samples, and 2 cm long segments were cut from parts of the leaves where the width was consistent. The total width was measured using a ruler to calculate the area (S). The segments were then dried and weighed (M1), and the remaining green leaves were also dried and weighed (M2) [30]. The formula for calculating LAI is:


$$\text{LAI}=\frac{\text{S}\times \text{N}\times (\text{M}1+\text{M}2)}{\text{A}\times \text{M}1}$$ (1)

The average LAI value of the four sample plants was calculated to represent the LAI of the small section.

#### AGB measurement.

For AGB measurement, four sample plants were collected from each small section and placed in a vacuum drying oven at a constant temperature of 100°C until they reached a constant weight (approximately 6 hours) [31]. The samples were then weighed on an electronic scale with a precision of 0.01 g. The average value of the four sample plants was taken as the AGB value for the small section.

#### Multi-spectral image data collection.

Multispectral images of the Gerbera at different growth stages across the entire experimental area were collected using a DJI Mavic 3M (DJI, Shenzhen, China). This device integrates four sensors with different central wavelengths (green: 560 ± 16 nm, red: 650 ± 16 nm, red edge: 730 ± 16 nm, near-infrared: 840 ± 26 nm), with a resolution of 2594*1944 pixels. The frequency of multispectral image collection was synchronized with the ground data collection, and flights were conducted between 9:00 and 15:00 on the same day, before the ground data collection. To reduce light interference within the greenhouse, the shading curtains were opened before the flight to ensure uniform lighting across all areas. During the acquisition of multispectral images, the UAV was manually flown. Based on the height of the greenhouse, the safe flying distance for the UAV, and the efficiency of image collection, the flight altitude was maintained at 3 meters with a spatial resolution of 1.57 cm, ensuring an image overlap greater than 80%. This was done to ensure the acquisition of complete high-resolution multispectral images.

### Data processing

#### Multi-spectral image processing.

After the multispectral image data collection, images from each of the four bands collected during each flight were selected according to the number of collections. Using DJI Terra 4.0.1 (which includes image correction and aerial triangulation optimization), 2D reconstructions of visible light images and Vegetation Index (VIs) images were generated for each collection. A total of five VIs images were generated for each collection, as shown in Table 1: Normalized Difference Vegetation Index (NDVI) images, Green Normalized Difference Vegetation Index (GNDVI) images, Chlorophyll Index (LCI) images, Normalized Difference Red Edge (NDRE) images, and Optimized Soil-Adjusted Vegetation Index (OSAVI) images. After generating the VIs images, they were cropped based on the predefined experimental area, resulting in 24 images corresponding to the 24 small sections of the experimental area. Over the entire growing cycle, a total of 840 VIs images (NDVI, GNDVI, LCI, NDRE, OSAVI) were obtained, which were used for the next stage of feature extraction.

**Table 1:** Introduction to the five VIs text.

Vegetation Index (VIs)	Formula^a^	Reference
**Normalized Difference Vegetation Index (NDVI)**	$\text{NDVI}=\frac{\text{NIR}-\text{R}}{\text{NIR}+\text{R}}$	[32]
**Green Normalized Difference Vegetation Index (GNDVI)**	$\text{GNDVI}=\frac{\text{NIR}-\text{G}}{\text{NIR}+\text{G}}$	[33]
**Leaf Chlorophyll Index (LCI)**	$\text{LCI}=\frac{\text{NIR}-\text{R}}{\text{NIR}+\text{R}}$	[34]
**Normalized Difference Red Edge Index (NDRE)**	$\text{NDRE}=\frac{\text{RE}-\text{NIR}}{\text{RE}+\text{NIR}}$	[35]
**Optimized Soil-Adjusted Vegetation Index (OSAVI)**	$\text{OSAVI}=\frac{(\text{NIR}+\text{C})-\text{R}}{(\text{NIR}+\text{C})+\text{R}}$	[36]

^a^G represents the reflectance in the green band, R represents the reflectance in the red band, RE represents the reflectance in the red edge band, NIR represents the reflectance in the near-infrared band, C is a constant, typically set to 0.16, used to reduce the impact of soil background.

#### Fusion feature extraction.

The fusion features in this study are based on a deep integration of VIs images with texture features. The Gray-Level Co-occurrence Matrix (GLCM) method was used to calculate Contrast, Homogeneity, Energy, Correlation, and Entropy [37] for the five VIs images. These features help represent the complexity of the leaf surface structure, health status, and boundaries between plants. In this experiment, calculations were performed using the skimage package in Python. Firstly, the pixel values of the images were linearly scaled to a range between 0 and 255. Then, the GLCM was calculated for adjacent pixels at angles of horizontal, 45 degrees to the upper right, vertical, and 135 degrees to the upper left, with the image grayscale levels set to 256. Finally, the five features of the GLCM were averaged. A total of 840 fusion feature samples of texture features were extracted from five VIs during three growth stages of Gerbera.

#### Data preprocessing.

To convert the 840 fusion features and growth indicators to the same scale, we used the Z-Score method to eliminate unit and scale differences before modeling. This method also helps detect and handle potential outliers in the dataset. Z-Score is a statistical measure that describes how many standard deviations an element is from the mean. It is calculated by subtracting the mean from the value and dividing by the standard deviation [38].The formula for Z-Score is as follows:


$$Z=\frac{\left(X-\mu \right)}{\sigma }$$ (2)

Where X is the data point, μ is the mean of all data points, σ is the standard deviation, and Z is the Z-Score value [39].

In this study, Z-Score was calculated for each column of data to detect outliers (using a default threshold of 3; if the absolute value of a data point’s Z-Score exceeds 3, it is considered an outlier). These outliers were then replaced with the median of that column to mitigate their impact on the analysis. After handling the outliers, Z-Score normalization was performed on the processed data. This converts the data into a form where the mean is 0 and the standard deviation is 1, facilitating subsequent data analysis or model training. An example of the processed data after Z-Score normalization is shown in Table 2. We obtained 840 data points, including five fusion features and growth indicators, for subsequent analysis and modeling.

**Table 2:** Example data of fusion features and growth indicators after Z-Score normalization.

VIs	Contrast	Homogeneity	Energy	Correlation	Entropy	PH	SPAD	LAI	AGB
**GNDVI**	-1.1634	-0.3271	-0.6010	0.4568	0.1466	-0.5717	0.3799	-0.4978	-0.5095
	-0.8745	-0.8051	-0.6461	-0.6082	0.4250	-2.1119	0.1620	-0.5232	-0.7095
	-0.9279	-0.7334	-0.6436	-0.2908	0.4151	-0.0491	-0.1804	-0.5486	-0.9096
	...	...	...	...	...	...	...	...	
	-1.4708	0.8779	-0.4544	1.5228	-0.6033	1.6286	1.5784	-0.2859	-0.0803
	-1.4848	1.0691	-0.3857	1.4605	-0.7699	1.7661	1.5628	3.8472	2.1332
	-1.4639	0.7912	-0.4590	1.3514	-0.6024	1.8761	1.3294	-0.2859	-0.0803
**LCI**	2.5730	-1.0434	-0.3725	-1.2829	1.0152	-0.5717	0.3799	-0.4978	-0.5095
	1.9506	0.0830	0.6229	-0.9884	-0.3175	-2.1119	0.1620	-0.5232	-0.7095
	1.4394	0.4499	0.8453	-0.5486	-0.5430	-0.0491	-0.1804	-0.5486	-0.9096
	...	...	...	...	...	...	...	...	
	-0.7845	0.0481	-0.1900	1.6494	0.2856	1.6286	1.5784	-0.2859	-0.0803
	-0.8674	-0.0393	-0.4329	1.6099	0.3800	1.7661	1.5628	3.8472	2.1332
	-0.5257	-0.2072	-0.3326	1.4573	0.5494	1.8761	1.3294	-0.2859	-0.0803
**NDRE**	0.5116	-0.7002	-0.2976	0.1344	0.7455	-0.5717	0.3799	-0.4978	-0.5095
	0.4997	0.3800	0.6854	-0.1057	-0.5198	-2.1119	0.1620	-0.5232	-0.7095
	0.1105	0.8384	1.0392	0.1627	-0.8880	-0.0491	-0.1804	-0.5486	-0.9096
	...	...	...	...	...	...	...	...	...
	-0.8567	-0.1152	-0.3637	1.5830	0.3598	1.6286	1.5784	-0.2859	-0.0803
	-0.9249	-0.1579	-0.5116	1.5024	0.4140	1.7661	1.5628	3.8472	2.1332
	-0.6417	-0.1897	-0.3177	1.4320	0.4916	1.8761	1.3294	-0.2859	-0.0803
**NDVI**	-0.4815	-0.7945	-0.6546	0.5900	0.8706	-0.5717	0.3799	-0.4978	-0.5095
	1.5808	-1.3483	-0.7173	-0.4074	1.5149	-2.1119	0.1620	-0.5232	-0.7095
	1.0187	-1.2243	-0.7035	-0.0195	1.4040	-0.0491	-0.1804	-0.5486	-0.9096
	...	...	...	...	...	...	...	...	...
	-1.2327	0.2804	-0.5324	1.7283	0.1950	1.6286	1.5784	-0.2859	-0.0803
	-1.2691	0.5877	-0.4204	1.6915	-0.0954	1.7661	1.5628	3.8472	2.1332
	-1.1671	0.1385	-0.5429	1.6163	0.2893	1.8761	1.3294	-0.2859	-0.0803
**OSAVI**	-0.8037	-0.7139	-0.6791	0.7566	0.6052	-0.5717	0.3799	-0.4978	-0.5095
	-0.5581	-0.8442	-0.6695	0.3302	0.5541	-2.1119	0.1620	-0.5232	-0.7095
	-0.5835	-0.7616	-0.6518	0.5098	0.4679	-0.0491	-0.1804	-0.5486	-0.9096
	...	...	...	...	...	...	...	...	...
	-1.0827	0.4645	-0.5622	1.4645	-0.0502	1.6286	1.5784	-0.2859	-0.0803
	-1.1092	0.4583	-0.5682	1.5039	-0.0304	1.7661	1.5628	3.8472	2.1332
	-1.0341	0.3469	-0.5648	1.3900	-0.0338	1.8761	1.3294	-0.2859	-0.0803

#### Model evaluation metrics.

In this study, the Coefficient of Determination (R^2^) and Root Mean Square Error (RMSE) were chosen to evaluate the accuracy of model training and validation. The R^2^ represents the proportion of the variance in the dependent variable that is predictable from the independent variable. The formula for R^2^ is as follows:


$$R^{2}=1-\frac{\sum_{i=1}^{n}{\left(y_{i}-{\hat{y}}_{i}\right)}^{2}}{\sum_{i=1}^{n}{\left(y_{i}-\overset{―}{y}\right)}^{2}}$$ (3)

where $y_{i}$ is the observed value, ${\hat{y}}_{i}$ is the predicted value, is the mean of the observed values, and n is the number of observations [40]. The value of R^2^ ranges from 0 to 1, where 0 indicates that the model does not explain any of the variability in the data, and 1 indicates that the model perfectly explains all the variability in the data.

The RMSE is a measure of the differences between the predicted and actual values. It provides a standard size of prediction error and has the same units as the original data, making it easy to understand and interpret. The formula for RMSE is as follows:


$$\mathrm{\backslash [}RMSE=\sqrt{\frac{1}{n}\sum_{i=1}^{n}{\left(y_{i}-{\hat{y}}_{i}\right)}^{2}}\mathrm{\backslash ]}$$ (4)

where $y_{i}$ is the observed value, ${\hat{y}}_{i}$ is the predicted value, and n is the number of observations [40]. In model evaluation, a smaller RMSE value indicates better model performance.

#### Construction of the OBM-RFEcv adaptive ensemble model.

In this study, we extracted five texture features (Contrast, Homogeneity, Energy, Correlation, and Entropy) from the five VIs images (NDVI, GNDVI, LCI, NDRE, OSAVI) using the GLCM method. These features were used to build the models. Six machine learning regression algorithms were selected as base models and comparison models: Linear Regression (LR) [41], Decision Tree Regression (DTR) [42], Random Forest Regression (RFR) [43], Gradient Boosting Regression (GBR) [44], Extreme Gradient Boosting Regression (XGBR) [45], and Support Vector Regression (SVR) [46]. First, the fusion features were used as model inputs to construct six base models: LR, DTR, RFR, GBR, XGBR, and SVR. During the model training and evaluation phase, 5-fold cross-validation was incorporated to ensure model stability. After calculating the R^2^ and RMSE for these six base models, the Python ‘max’ function was used to automatically select the base model with the highest R^2^ value as the Optimal Base Model (OBM). This OBM also serves as the base model for the ensemble model.

**Fig 2:**
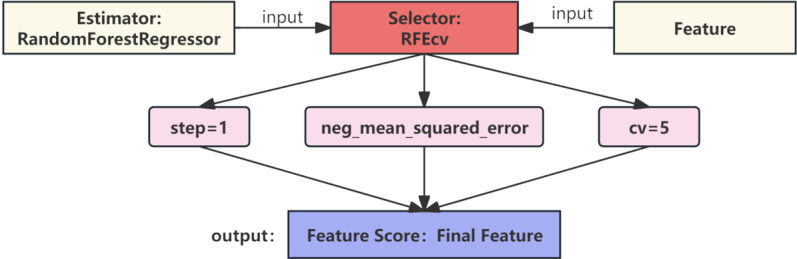
RFEcv algorithm flowchart.

To recursively remove the least important features during model training, we used the cross-validated version of Recursive Feature Elimination (RFEcv) for feature selection. RFEcv repeatedly builds models and retains the best features until a predetermined number of features is reached. The core idea of RFE is to judge the importance of features based on model weights and gradually eliminate less important features to optimize model performance [47]. Compared to RFE, RFEcv can automatically determine the optimal number of features through cross-validation. The RFEcv algorithm process is shown in Fig 2. Since RFEcv is model-based, it can be integrated into various types of models [48].

As shown in Fig 2, in this experiment, the Random Forest Regressor algorithm was used as the estimator for RFEcv. In each iteration, one feature was removed, and 5-fold cross-validation was used to determine the optimal number of features. The negative mean squared error (NMSE) of each feature was calculated as the evaluation metric for automatic feature selection.

**Fig 3:**
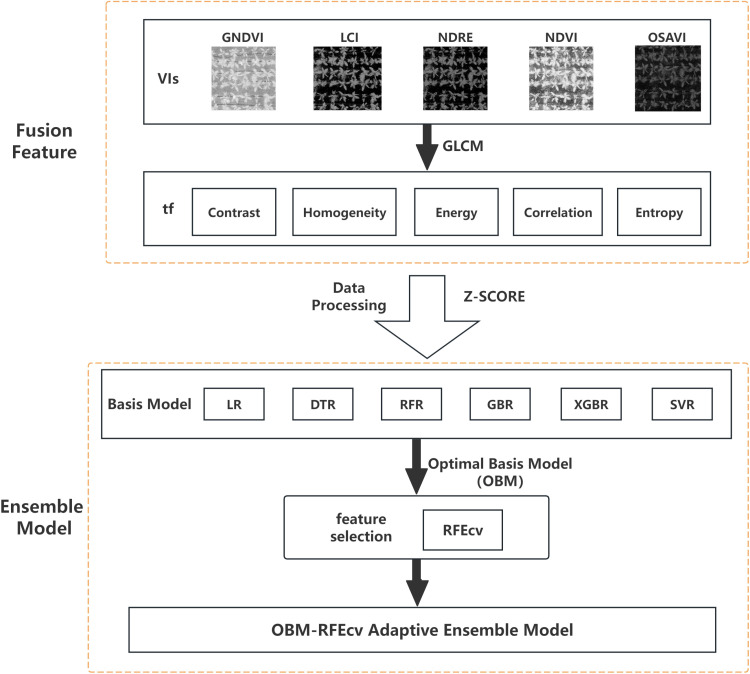
Technical route diagram.

After selecting the OBM, we integrated the RFEcv algorithm into the OBM to form an adaptive ensemble model, OBM-RFEcv, for monitoring the growth indicators of Gerbera. The technical route of this study is shown in Fig 3.

## Results

This study developed monitoring models for the growth stages (growth, early flowering, and full bloom) of Gerbera, specifically for PH, SPAD, LAI, and AGB, based on the fusion features extracted from low-altitude UAV multispectral images, including VIs and texture features. We used the fusion feature variables extracted from five VIs images across three growth stages of Gerbera to build 140 monitoring models (4*5*7) for four growth indicators using seven models. We compared and analyzed the fusion features of five different VIs images for PH, SPAD, LAI, and AGB in Gerbera using six base models and the OBM-RFEcv adaptive ensemble model. The performance of the base models and the adaptive ensemble model was compared, and the model performance was tested on a test set using R^2^ and RMSE as evaluation metrics. Regarding the dataset division, 80% of the samples were used for training and 20% for testing in this experiment. For model parameters, n_estimators = 100 and random_state = 42 were set, with all other parameters being default values. The cross-validation was performed using 5-fold cross-validation.

### Monitoring results of Gerbera PH indicators with different VIs image fusion features

As shown in Table 3, the combination of the OBM-RFEcv model and the NDVI fusion feature performed best in the PH indicator monitoring results, with R^2^ and RMSE values of 0.92 and 0.04, respectively. Compared to the optimal base models RFR and GBR, the R^2^ value increased by 0.08, and the RMSE decreased by 0.01. Although the base models did not perform optimally with NDVI compared to other VIs, the accuracy improved significantly after incorporating RFEcv, with R^2^ values 0.2 to 0.5 higher and the highest RMSE reduction of 0.2. For OSAVI, although the base models already showed high accuracy, the inclusion of RFEcv reduced the accuracy, indicating that the fusion features based on OSAVI do not require feature selection, but the accuracy is still lower than the combination of OBM-RFEcv and NDVI.

**Table 3:** Evaluation results of Gerbera PH indicators with different models and VIs.

Model	GNDVI		LCI		NDRE		NDVI		OSAVI	
	R2	RMSE	R2	RMSE	R2	RMSE	R2	RMSE	R2	RMSE
**LR**	0.03	0.16	0.77	0.07	0.84	0.06	0.79	0.06	0.82	0.07
**DTR**	0.74	0.07	0.76	0.07	0.78	0.07	0.65	0.08	0.90	0.06
**RFR**	0.72	0.07	0.83	0.06	0.88	0.05	0.84	0.05	0.91	0.05
**GBR**	0.77	0.06	0.83	0.06	0.88	0.05	0.84	0.05	0.91	0.05
**XGBR**	0.56	0.08	0.80	0.06	0.83	0.06	0.83	0.05	0.90	0.06
**SVR**	0.03	0.16	0.77	0.07	0.82	0.06	0.72	0.07	0.81	0.08
**OBM-RFEcv**	0.87	0.04	0.88	0.05	0.90	0.05	0.92	0.04	0.87	0.06

**Fig 4:**
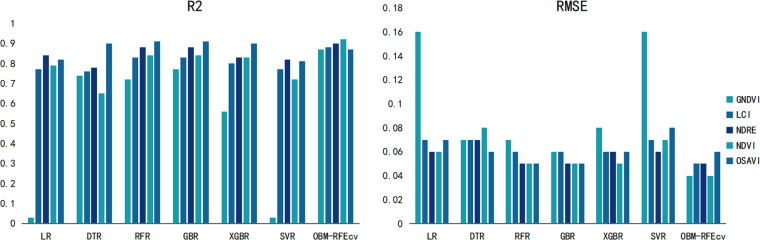
Comparison of evaluation results of gerbera PH indicators with different models and VIs.

From Fig 4, it can also be seen that the OBM-RFEcv model, compared to the base models, not only showed a significant improvement in R^2^ and RMSE but also made the evaluation results of the fusion features of the five different VIs more balanced. The most significant improvement was observed with the NDVI feature, indicating that the combination of the OBM-RFEcv model and the NDVI fusion feature is the most effective in capturing and monitoring plant height. Based on the PH monitoring model with higher R^2^ and lower RMSE, managers can better understand the crop’s growth rate and developmental stages. Accurate PH data can also assist in planning more reasonable planting layouts and densities.

**Fig 5:**
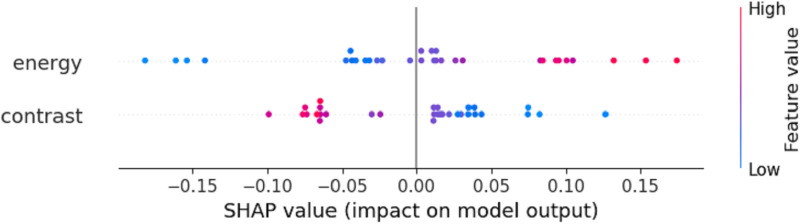
SHAP plot of the PH monitoring model.

Fig 5 illustrates the impact of two texture features, Energy and Contrast, on the model’s prediction results after selection by the OBM-RFEcv model. For the Energy feature, most points are located on the right side, with more red points than blue ones, while the left side is mostly blue points. As the feature value increases (from blue to red), its positive contribution to the model’s prediction gradually strengthens. For the Contrast feature, the points are mainly concentrated on the left side, with colors ranging from red to purple, indicating a significant negative contribution to the model’s prediction. As the feature value increases (from red to purple), its negative contribution to the model’s prediction gradually decreases.

### Monitoring results of Gerbera SPAD indicators with different VIs image fusion features

According to the SPAD monitoring results in Table 4, the OBM-RFEcv model also showed higher accuracy than the base models, especially when combined with the GNDVI, where R^2^ and RMSE were 0.90 and 0.07, respectively. Compared to the optimal base model GBR, the R^2^ value increased by 0.03, and the RMSE decreased by 0.01. Compared to other VIs, the R^2^ value increased by 0.02 to 0.17, and the RMSE decreased by up to 0.03. Similarly, in the GNDVI, the average performance of the base models was better than in other VIs, indicating that the GNDVI feature is more sensitive to SPAD in Gerbera. The lowest performance was observed with the LCI, with R^2^ and RMSE values of 0.73 and 0.10, respectively, indicating a lower correlation between SPAD and the LCI index.

**Table 4:** Evaluation results of Gerbera SPAD indicators with different models and VIs.

Model	GNDVI		LCI		NDRE		NDVI		OSAVI	
	R2	RMSE	R2	RMSE	R2	RMSE	R2	RMSE	R2	RMSE
**LR**	0.75	0.12	0.52	0.17	0.75	0.11	0.33	0.19	0.61	0.14
**DTR**	0.77	0.11	0.48	0.17	0.73	0.11	0.59	0.14	0.62	0.14
**RFR**	0.85	0.09	0.71	0.12	0.79	0.10	0.70	0.12	0.82	0.09
**GBR**	0.87	0.08	0.70	0.12	0.79	0.10	0.74	0.12	0.80	0.10
**XGBR**	0.83	0.09	0.68	0.13	0.75	0.11	0.62	0.14	0.77	0.10
**SVR**	0.74	0.12	0.49	0.17	0.75	0.11	0.19	0.21	0.60	0.14
**OBM-RFEcv**	0.90	0.07	0.73	0.10	0.88	0.08	0.82	0.09	0.87	0.08

**Fig 6:**
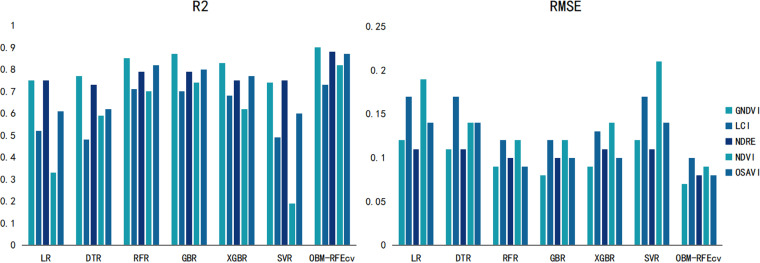
Comparison of evaluation results of Gerbera SPAD indicators with different models and VIs.

Based on Fig 6, it is evident that the OBM-RFEcv model achieved a significant increase in R^2^ compared to the base models for all five VIs image fusion features, and the RMSE values were the lowest. The performance of all fusion features was relatively balanced, with the most significant improvement observed in GNDVI. Higher R^2^ and lower RMSE allow managers to gain a deeper understanding of the crop’s health status and nutrient absorption efficiency. By monitoring changes in SPAD values, it is possible to quickly detect whether crops suffer from nutrient deficiencies such as nitrogen deficiency. Accurate SPAD values can help determine the optimal timing for fertilization, ensuring that necessary nutrients are provided precisely when the crops need them most, thereby promoting smooth transitions through all growth stages.

**Fig 7:**
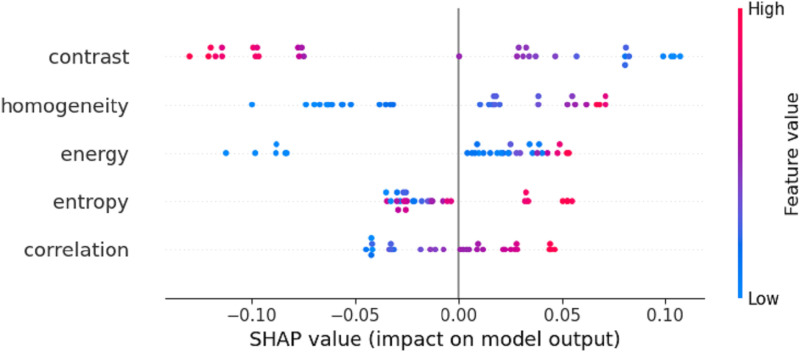
SHAP plot of the SPAD monitoring model.

Fig 7 illustrates the impact of five selected texture features—Contrast, Homogeneity, Energy, Entropy, and Correlation—on the model’s prediction results after selection by the OBM-RFEcv model. Except for the Entropy feature, the points for the other four features are concentrated on the right side, indicating that these four features generally have a positive contribution to the model’s predictions. Conversely, the Entropy feature shows an opposite trend. As the value of the Contrast feature increases (from blue to red), its negative contribution to the model’s prediction gradually strengthens. In Contrast, for the other four features (Homogeneity, Energy, Correlation), as their values increase (from blue to red), their positive contributions to the model’s prediction gradually strengthen. Notably, the Homogeneity feature exhibits a higher positive contribution compared to the other three features (Energy, Correlation, and Contrast).

### Monitoring results of Gerbera LAI indicators with different VIs image fusion features

As shown in Table 5, the OBM-RFEcv model combined with the GNDVI performed better and had higher accuracy than other combinations for LAI monitoring. The R^2^ and RMSE values were 0.89 and 0.08, respectively, which were 0.12 higher in R^2^ and 0.02 lower in RMSE compared to the optimal base model XGBR. In the OBM-RFEcv model, the R^2^ value for the GNDVI feature was 0.01 to 0.14 higher than for other VI combinations, and the RMSE values also showed good performance, decreasing by 0.01 to 0.04. The worst performance was observed with the OSAVI, indicating that it is not sensitive enough to the LAI of Gerbera, and there were significant differences in the performance of different models.

**Table 5:** Evaluation results of Gerbera LAI indicators with different models and VIs.

Model	GNDVI		LCI		NDRE		NDVI		OSAVI	
	R2	RMSE	R2	RMSE	R2	RMSE	R2	RMSE	R2	RMSE
**LR**	0.65	0.11	0.71	0.10	0.51	0.13	0.31	0.15	0.10	0.18
**DTR**	0.69	0.10	0.85	0.06	0.43	0.15	0.64	0.10	0.66	0.10
**RFR**	0.75	0.09	0.88	0.06	0.69	0.11	0.68	0.10	0.76	0.09
**GBR**	0.71	0.10	0.88	0.06	0.61	0.12	0.61	0.10	0.73	0.09
**XGBR**	0.77	0.09	0.87	0.06	0.52	0.13	0.49	0.12	0.68	0.10
**SVR**	0.56	0.12	0.72	0.10	0.51	0.14	0.46	0.13	0.27	0.17
**OBM-RFEcv**	0.89	0.08	0.88	0.09	0.84	0.10	0.77	0.12	0.75	0.09

**Fig 8:**
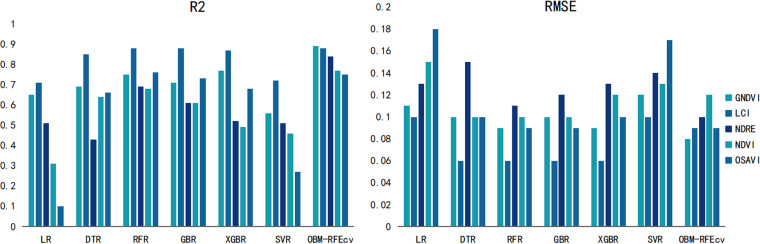
Comparison of evaluation results of Gerbera LAI indicators with different models and VIs.

From Fig 8, it can also be seen that the GNDVI showed significantly better R^2^ and RMSE values than other indices, and the OBM-RFEcv model’s performance was more accurate than the base models. This indicates that the OBM-RFEcv model has good generalization ability for different VIs features, and combining it with the GNDVI is more suitable for predicting the LAI of Gerbera. In LAI monitoring, higher R^2^ and lower RMSE allow managers to more accurately understand changes in crop canopy structure and photosynthesis efficiency. Changes in LAI can serve as early warning signals, indicating potential pest infestations or nutrient imbalances.

**Fig 9:**
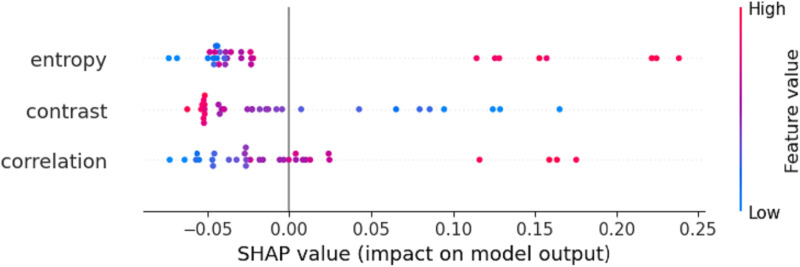
SHAP plot of the LAI monitoring model.

Fig 9 illustrates the impact of three selected texture features—Entropy, Contrast, and Correlation—on the model’s prediction results after selection by the OBM-RFEcv model. The points for these three features are mostly concentrated on the left side, indicating that most of their feature values have a slight negative contribution to the model’s predictions.As the feature values increase (from blue to red), the positive contributions of the Entropy and Correlation features to the model’s predictions gradually strengthen. Among them, the Entropy feature has a greater positive contribution compared to the Correlation feature. Conversely, the Contrast feature shows an opposite trend, where its negative contribution gradually strengthens as its feature values increase.

### Monitoring results of Gerbera AGB indicators with different VIs image fusion features

From Table 6, the OBM-RFEcv model combined with the NDRE performed best in AGB monitoring, with R^2^ and RMSE values of 0.93 and 0.07, respectively. The R^2^ value was the highest among all growth indicators. Compared to the optimal base model RFR, the R^2^ value of the OBM-RFEcv model increased by 0.11, and the RMSE decreased by 0.04, showing a significant improvement. Although the base models did not perform optimally with the NDRE index, the accuracy improved significantly after using the OBM-RFEcv model, indicating that not all features are sensitive to AGB in the NDRE, and feature selection is necessary.

**Table 6:** Evaluation results of Gerbera AGB indicators with different models and VIs.

Model	GNDVI		LCI		NDRE		NDVI		OSAVI	
	R2	RMSE	R2	RMSE	R2	RMSE	R2	RMSE	R2	RMSE
**LR**	0.78	0.12	0.67	0.15	0.73	0.14	0.41	0.22	0.64	0.15
**DTR**	0.75	0.13	0.52	0.16	0.76	0.12	0.30	0.22	0.70	0.14
**RFR**	0.84	0.10	0.63	0.14	0.82	0.11	0.60	0.17	0.77	0.11
**GBR**	0.84	0.10	0.56	0.15	0.76	0.12	0.50	0.18	0.72	0.13
**XGBR**	0.83	0.10	0.63	0.14	0.81	0.11	0.30	0.22	0.67	0.12
**SVR**	0.74	0.13	0.67	0.15	0.75	0.13	0.38	0.22	0.73	0.15
**OBM-RFEcv**	0.83	0.13	0.89	0.09	0.93	0.07	0.80	0.12	0.79	0.12

**Fig 10:**
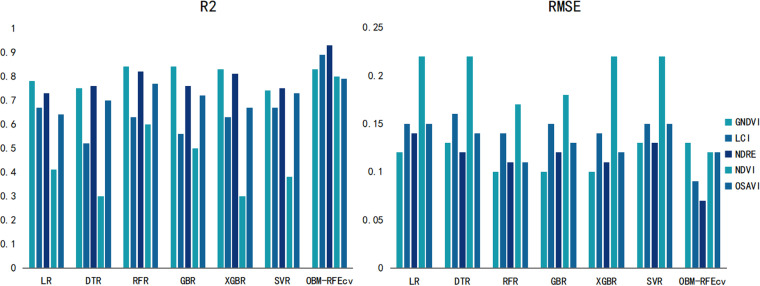
Comparison of evaluation results of Gerbera AGB indicators with different models and VIs.

In Fig 10, the OBM-RFEcv model performed best among all VIs image features, and the results of all features based on the OBM-RFEcv model were more balanced, indicating that the OBM-RFEcv model can significantly improve the prediction accuracy of various vegetation features, with stronger overall performance, making it very suitable for monitoring the AGB of Gerbera. By accurately assessing the biomass accumulation of crops, managers can adjust fertilization plans according to the actual needs of the crops, promoting healthy growth while reducing the use of chemical fertilizers and minimizing environmental impact. Utilizing improved AGB monitoring results, farmers can predict the final crop yield in advance.

**Fig. 11:**
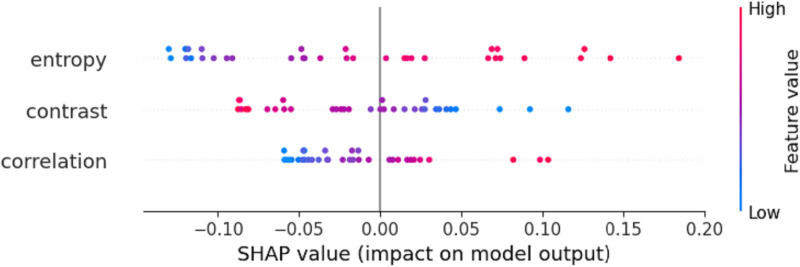
SHAP plot of the AGB monitoring model.

Fig 11 illustrates the impact of three selected texture features—Entropy, Contrast, and Correlation—on the model’s prediction results after selection by the OBM-RFEcv model. The points for these three features are mostly concentrated on the left side, indicating that most of their feature values have a negative contribution to the model’s predictions.

As the feature values increase (from blue to red), the positive contributions of the Entropy and Correlation features to the model’s predictions gradually strengthen. Similar to the LAI monitoring model, the Entropy feature has a greater positive contribution compared to the Correlation feature. Conversely, the Contrast feature shows an opposite trend, where its negative contribution to the model’s predictions gradually strengthens as its feature values increase.

## Discussion

### Impact of fusion features based on different VIs images on estimating Gerbera growth indicators

By utilizing high-resolution multispectral images obtained from UAVs, we extracted five texture features—Contrast, Homogeneity, Energy, Correlation, and Entropy—from five VIs images using the GLCM technique. These features, when combined with traditional VIs, provided a richer information source for estimating PH, SPAD, LAI, and AGB in Gerbera. Wei et al. [49] monitored chlorophyll content in winter wheat canopies under different nitrogen treatments using UAV multispectral images, selecting 16 spectral vegetation indices to establish SPAD estimation models for different growth stages, with an R^2^ of 0.73 in the optimal stepwise regression model. Guomin et al. [50] used five VIs as input parameters and employed the random forest regression algorithm to establish the relationship between canopy vegetation indices and LAI for field maize under different irrigation conditions, with an R^2^ of 0.87. Guo et al. [51] used images acquired from a multispectral imaging spectrometer mounted on a UAV and extracted plant height from Digital Surface Model (DSM) information to establish an improved AGB estimation model using back propagation neural networks, with an R^2^ of 0.88. Despite the innovations in spectral data processing and the relatively high model accuracy achieved in the aforementioned studies, most of them rely solely on vegetation indices or directly use raw spectral data for crop growth status assessment during feature extraction. Additionally, these studies do not employ model ensemble techniques in model construction, which may result in weaker generalization ability and poorer applicability of the models. In contrast, this study employs a feature fusion strategy that extracts texture features from VI images and constructs an adaptive ensemble model. This approach can more accurately capture subtle changes during the plant growth process and enhances the model’s generalization ability.

Additionally, by using various regression models to select the fusion features extracted from different VIs images, we further ensured that the selected features have strong correlations with Gerbera growth indicators. This approach not only effectively captures the complex relationships between different VIs and target variables, improving model prediction accuracy, but also reduces unnecessary computational burden. For economic crops like Gerbera, rapid and accurate monitoring of growth status is crucial for optimizing field management decisions.

### Constructing the adaptive ensemble model OBM-RFEcv to improve overall model performance

To determine the effectiveness of the adaptive ensemble model OBM-RFEcv in monitoring key growth indicators of Gerbera, we compared the performance of multiple single regression models, including LR, DTR, RFR, GBR,XGBR and SVR. The experimental results showed that while some single models performed well under specific conditions, their overall predictive capabilities were limited.

In contrast, the proposed OBM-RFEcv adaptive ensemble model, which combines the feature selection method RFEcv with the optimal model among the various regression models, improved the estimation accuracy of Gerbera PH, SPAD, LAI, and AGB across different VIs, showing better overall performance. Especially in estimating above-ground biomass, the OBM-RFEcv + NDRE combination achieved R^2^ = 0.93 and RMSE = 0.07, significantly outperforming the best-performing model without feature selection. This indicates that the RFEcv method, by recursively removing the least important features and retraining the model, is capable of identifying the most predictive subset of features. This approach not only reduces model complexity but also avoids overfitting, making the model more efficient and accurate when handling complex nonlinear relationships. The OBM-RFEcv enhances this by adaptively selecting the optimal base models for ensemble learning, improving the fitness of different data across various models, thereby enhancing overall predictive capability. This strategy is particularly suitable for agricultural data characterized by high heterogeneity and complexity, aiding in capturing subtle changes under different growth stages and environmental conditions.Future research could further expand the number of base models to better match different datasets, improving the universality and practicality of the models.

Whether for field crops or economic crops, research related to the adaptive ensemble model strategy is still relatively scarce. Although the adaptive ensemble model proposed in this study has shown good performance and has been tested on multiple varieties of Gerbera, the number of varieties covered by the current study remains limited compared to the rich diversity of Gerbera species. Significant genetic differences, morphological characteristics, and varying responses to external environments among different varieties may affect the prediction accuracy of the model. In addition to increasing the number of Gerbera varieties, it is also necessary to validate the model on other types of economic crops to test its generalizability across a broader range of crop types.

Even though the model has been tested on various Gerbera varieties, these tests were mostly conducted within specific geographic regions and under relatively consistent planting conditions. Different climatic zones, soil conditions, and agricultural management practices can have significant impacts on crop growth. Therefore, testing the model’s performance in more diverse environments is essential.

In addition to utilizing data acquired via UAVs, future work could consider integrating information from other sources. Combining with micro-environmental data such as soil moisture and temperature provided by ground sensor networks could offer richer input information for crop growth models. For instance, real-time monitoring of soil moisture can help adjust irrigation schedules, while temperature data can assist in predicting the risk of pest and disease outbreaks. Integrating data from meteorological stations, such as precipitation, wind speed, and light intensity, could further refine crop growth models, enabling them to better reflect the impact of external environments on crops. This comprehensive approach would lead to the construction of more thorough and precise models for monitoring the crop growth process.

## Conclusions

This study explored the impact of fusion features based on different VIs images and texture features on the monitoring performance of Gerbera growth indicators. The OBM-RFEcv adaptive ensemble model was used to analyze and evaluate the monitoring results of Gerbera PH, SPAD, LAI, and AGB, and comparisons were made with base models.

The results showed that the OBM-RFEcv model exhibited high accuracy in estimating Gerbera PH, SPAD, LAI, and AGB, yielding good prediction results for fusion features of all VIs images. Compared to the base models, the OBM-RFEcv model achieved an approximately 2% improvement in R^2^ and RMSE, with more balanced performance. Specifically, the OBM-RFEcv model performed best in monitoring PH with NDVI, achieving R^2^ = 0.92 and RMSE = 0.04; in monitoring SPAD, GNDVI performed best, with R^2^ = 0.90 and RMSE = 0.07; in monitoring LAI, GNDVI performed best, with R^2^ = 0.89 and RMSE = 0.08; and finally, in monitoring AGB, NDRE performed best, with R^2^ = 0.93 and RMSE = 0.07. In summary, the feature fusion method based on extracting texture features from different VIs images has high application potential and practicality for monitoring growth indicators during the growth stages of Gerbera. The OBM-RFEcv model demonstrated more balanced and superior performance than the base models across different VIs image fusion features. The advantage of the OBM-RFEcv model lies in its use of the RFEcv feature selection method to optimize input variables, reducing the influence of irrelevant features and thus improving prediction accuracy. Furthermore, the OBM-RFEcv model more effectively captured the complex relationships between different VIs and growth indicators, significantly enhancing the model’s generalization ability, particularly under NDVI, GNDVI, and NDRE. These findings provide important references for future research and applications in monitoring the growth indicators of economic crops in greenhouse environments using low-altitude multispectral UAVs.

## Supporting information

S1 File The original data used in this study. (XLSX)
